# The Ensemble Mars Atmosphere Reanalysis System (EMARS) Version 1.0

**DOI:** 10.1002/gdj3.77

**Published:** 2019-08-23

**Authors:** Steven J. Greybush, Eugenia Kalnay, R. John Wilson, Ross N. Hoffman, Thomas Nehrkorn, Mark Leidner, Janusz Eluszkiewicz, Hartzel E. Gillespie, Matthew Wespetal, Yongjing Zhao, Matthew Hoffman, Patrick Dudas, Timothy McConnochie, Armin Kleinböhl, David Kass, Daniel McCleese, Takemasa Miyoshi

**Affiliations:** ^1^ Department of Meteorology and Atmospheric Science The Pennsylvania State University University Park PA USA; ^2^ Institute for CyberScience The Pennsylvania State University University Park PA USA; ^3^ Department of Atmospheric and Oceanic Science The University of Maryland College Park MD USA; ^4^ NASA Ames Moffett Field CA USA; ^5^ Atmospheric and Environmental Research Verisk Analytics Lexington MA USA; ^6^ Rochester Institute of Technology Rochester NY USA; ^7^ Department of Astronomy The University of Maryland College Park MD USA; ^8^ Jet Propulsion Laboratory California Institute of Technology Pasadena CA USA; ^9^ Synoptic Science Altadena CA USA; ^10^ RIKEN Kobe Japan

**Keywords:** assimilation, atmosphere, ensemble, mars, reanalysis

## Abstract

The Ensemble Mars Atmosphere Reanalysis System (EMARS) dataset version 1.0 contains hourly gridded atmospheric variables for the planet Mars, spanning Mars Year (MY) 24 through 33 (1999 through 2017). A reanalysis represents the best estimate of the state of the atmosphere by combining observations that are sparse in space and time with a dynamical model and weighting them by their uncertainties. EMARS uses the Local Ensemble Transform Kalman Filter (LETKF) for data assimilation with the GFDL/NASA Mars Global Climate Model (MGCM). Observations that are assimilated include the Thermal Emission Spectrometer (TES) and Mars Climate Sounder (MCS) temperature retrievals. The dataset includes gridded fields of temperature, wind, surface pressure, as well as dust, water ice, CO_2_ surface ice and other atmospheric quantities. Reanalyses are useful for both science and engineering studies, including investigations of transient eddies, the polar vortex, thermal tides and dust storms, and during spacecraft operations.

## 1 INTRODUCTION

A Mars atmosphere reanalysis provides a comprehensive estimate of the state and temporal evolution of the atmosphere, combining available spacecraft observations to be consistent with the physics and dynamics of a global climate model. A reanalysis consists of an extended, retrospective sequence of analyses, created using data assimilation, of gridded atmospheric fields of variables such as temperature, wind, surface pressure, dust and water ice cloud opacities. Reanalyses have been used extensively for terrestrial applications (e.g. Kalnay et al., 1996) using contemporary models and assimilation systems with in situ and remotely sensed observations spanning decades, and include complete earth‐system reanalyses (Bosilovich, Rixen, & Chaudhuri, 2013) as well as those assimilating only surface pressure data (Compo, Whitaker, & Sardeshmukh, 2006). The Mars Analysis Correction Data Assimilation (MACDA) reanalysis is the first to be created for an extraterrestrial atmosphere (Montabone et al., 2014). EMARS is the first ensemble reanalysis for Mars, spanning multiple years across the observational datasets of multiple spacecraft instruments.

Data assimilation for Mars has been previously demonstrated by several studies (Houben, 1999; Lee et al., 2011; Lewis & Read, 1995; Lewis, Read, Conrath, Pearl, & Smith, 2007; Navarro, Forget, Millour, & Greybush, 2014; Navarro et al., 2017; Steele et al., 2014; Zhang et al., 2001). The foundations for EMARS were laid with the observing system simulation experiments (OSSEs) of Hoffman et al., (2010), which demonstrated that simulated Martian observations can constrain the atmospheric state via ensemble data assimilation. Greybush et al. (2012) demonstrated this with real spacecraft observations, tuning the data assimilation system for optimal performance. Further studies demonstrated the synergy between reanalysis development and science investigations. Zhao, Greybush, Wilson, Hoffman, and Kalnay (2015) studied thermal tides in a predecessor to EMARS and found that tuning the assimilation window was essential for avoiding spurious resonances. Waugh et al. (2016) compared polar vortices across reanalyses and motivated the inclusion of a topographic gravity wave drag parameterization to more faithfully reproduce the polar vortex. Greybush, Gillespie, and Wilson (2019) studied transient eddies in reanalyses and examined the robustness of convergence upon unique synoptic states.

Section 2 describes the spacecraft observations, Mars global climate model and data assimilation used in the preparation of the dataset. Section 3 describes the dataset and its formatting in detail. Section 4 discusses access to the dataset and its visualization. Section 5 describes current and potential uses for the dataset. Section 6 outlines expected future developments for the dataset.

## 2 DATA PRODUCTION METHODS

### 2.1 Description of the EMARS observations

The Ensemble Mars Atmosphere Reanalysis System uses atmospheric observations remotely sensed by two instruments on spacecraft orbiting Mars. The first instrument is the Thermal Emission Spectrometer (TES), which operated on the Mars Global Surveyor (MGS) from MY 24‐27, or 1999–2004. The TES nadir retrievals (Smith, Pearl, Conrath, & Christensen, 2001), available from the Planetary Data System (PDS), provide twice‐daily (2 a.m. and 2 p.m. local time in the tropics) coverage of temperature, dust and water ice column opacity. Vertical coverage is from the surface to ~40 km in altitude on up to 21 vertical levels, but there are only 2–5 effective vertical degrees of freedom in the profiles with decreasing resolution at higher altitudes, as estimated from averaging kernels as in Eluszkiewicz et al. (2008). The PDS retrievals have unrealistic jumps in temperature climatology, associated with the change in spectral resolution. Instrument data quality is further discussed in Pankine (2015; 2016).

The second instrument is the Mars Climate Sounder (MCS; McCleese et al., 2007), operating on the Mars Reconnaissance Orbiter (MRO, Zurek & Smrekar, 2007) since MY 28 (2006). Unfortunately, there is no temporal overlap between TES and MCS observations. However, the two datasets have been found to show good agreement at seasons with little interannual variability (Shirley et al., 2015). MCS provides limb retrievals of temperature, dust and water ice vertical profiles (Kleinböhl et al., 2009). Profile retrievals of temperature typically make use of co‐located limb and nadir or off‐nadir measurements in order to improve vertical coverage in the lower atmosphere, and reach over 80 km in altitude. The original along‐track observation strategy provides twice‐daily observations (3 a.m. and 3 p.m. local time in the tropics). Starting in 2010, cross‐track observations (Kleinböhl, Wilson, Kass, Schofield, & McCleese, 2013) were added, providing 6 local times of day coverage. From 2010 to 2014, intervals of multi‐track (along + cross‐track) observations alternated with intervals of along‐track only sampling; after 2014, multi‐track sampling was used continuously. In order to avoid changes in reanalysis climatology due to changes in observing patterns, EMARS v1.0 only assimilates along‐track observations. MCS retrievals are provided on 105 vertical pressure levels, whereas the vertical weighting functions indicate an effective vertical resolution of 5 km. MCS version 5 retrievals (Kleinböhl, Friedson, & Schofield, 2017), that consider a 2D geometry to provide superior capabilities in regions of sharp temperature gradients, are used. MCS retrievals, due to their limb geometry, have reduced sensitivity to the lowest 5–10 km of the atmosphere, which has impacts on the ability to resolve lower atmosphere transient eddies compared to TES (see Section 5.2).

### 2.2 Description of the EMARS Model

The Ensemble Mars Atmosphere Reanalysis System uses the Geophysical Fluid Dynamics Laboratory (GFDL) Mars Global Climate Model (MGCM) for the numerical weather prediction component of the reanalysis. This model, using a dynamical core originally developed for the atmosphere of Earth, has been adapted to operate with Mars atmosphere physics (Wilson & Hamilton, 1996) and adapted to work in a data assimilation framework (Greybush et al., 2012; Hoffman et al., 2010). The GFDL MGCM has been used to examine tides and planetary waves (Hinson & Wilson, 2002; Hinson, Wilson, Smith, & Conrath, 2003; Wilson & Hamilton, 1996), the water cycle (Richardson & Wilson, 2002; Richardson, Wilson, & Rodin, 2002), the dust cycle (Basu, Richardson, & Wilson, 2004; Basu, Wilson, Richardson, & Ingersoll, 2006; Wilson & Kahre, 2009), the influence of topography (Richardson & Wilson, 2002) and cloud radiative effects (Hinson & Wilson, 2004; Kleinböhl et al., 2013; Wilson, 2011; Wilson, Lewis, Montabone, & Smith, 2008; Wilson, Neumann, & Smith, 2007). The dynamical core is finite‐volume (Lin, 2004); EMARS uses the latitude/longitude geometry, with grid spacing of 6 degrees longitude by 5 degrees latitude (60 × 36). The model contains 28 vertical levels, with 13 of these levels being in the lowest scale height (~10 km) of the atmosphere. The vertical coordinate is a hybrid sigma‐pressure coordinate, with terrain‐following sigma levels near the surface transitioning to pressure levels above 2 Pa. The vertical grid spacing increases substantially with height.

Model physics were adapted to the Martian atmosphere. The representation of dust is controlled by three radiatively active tracers, with particle radii of 0.3, 1.2 and 2.5 microns, that undergo advection and sedimentation. Radiatively active water ice clouds are employed in the MGCM. The MGCM has an active, multi‐phase CO_2_ cycle. When temperatures are projected to be below the (pressure‐dependent) CO_2_ critical temperature in the atmosphere, the gaseous CO_2_ mass needed to generate the appropriate latent heating is removed from the atmosphere and placed on the surface as CO_2_ snow. There is no explicit CO_2_ cloud microphysics. A parameterization for sub‐grid‐scale topographic gravity wave drag is employed, as in Waugh et al. (2016).

### 2.3 Description of the EMARS data assimilation system

The Ensemble Mars Atmosphere Reanalysis System uses an ensemble‐based data assimilation system, the Local Ensemble Transform Kalman Filter (LETKF; Hunt, Kostelich, & Szunyogh, 2007), developed at the University of Maryland and coded by Takemasa Miyoshi (https://github.com/takemasa-miyoshi/letkf). Data assimilation systems combine a background, or first guess, with observations to produce an analysis, which represents the best estimate of the state of the atmosphere. The update to the background (analysis increment) depends on the differences between the background and observations (observation increment); calculation of the observation increment is described in Section 2.3.2. The relative weighting of background and observation errors also determines the magnitude of the analysis increment; the details of this are described in Section 2.3.3. The spatial pattern of the analysis increment and the impact of one variable on another are described in Section 2.3.4. The advantage of an Ensemble Kalman Filter (EnKF) is that the background error covariance is sampled from a dynamical ensemble of simulations, and is therefore flow dependent (Kalnay, Li, Miyoshi, Yang, & Ballabrera, 2007). Further details on the equations used for data assimilation can be found in Greybush et al. (2012).

#### 2.3.1 Observation preprocessing

Temperature profile observations are then prepared for assimilation. Temporally, observations are collected from the 1‐hr interval centred on each hour, which is the time of the analysis. In order to match the scales resolved by the observations to those resolved by the model and reduce errors of representativeness and random instrumental errors, the raw TES and MCS observations are first preprocessed to create ‘superobservations’ (e.g. Alpert & Kumar, 2007). In the horizontal, observations are binned to the nearest model grid point, and the superobservation consists of the mean observation value, latitude and longitude in each bin. Horizontal resolution of observations, particularly TES, is greater than that of the model in the along‐track direction, whereas the superobservations have similar resolution. In the vertical, TES observations have only ~2–5 degrees of freedom in the vertical and MCS observations ~20. Therefore, the raw temperature profiles of 21 (TES) and 105 (MCS) vertical levels are averaged to reflect this (as in Montabone et al., 2014), effectively performing a vertical superobservation. Observation errors include instrument measurement error, forward model error and errors of representativeness. As estimates of the observation uncertainties were not provided with the TES observations in the PDS, EMARS v1.0 assigns an observation error of 3.0 K to the superobservations. MCS does include uncertainty estimates with its retrievals; these are used by EMARS.

#### 2.3.2 Observation operator

The observation operator (forward operator) maps (i.e. converts) the model background to simulated observations in ‘observation space’ (at the same locations, variable types as the observations). The observation increments are then the actual observations minus the simulated observations. In this version of EMARS, only temperature observations are directly assimilated, and therefore, the observation operator maps from model temperatures to retrieved temperatures. Model temperature fields are horizontally interpolated to observation locations. Model vertical profiles are then interpolated to the same pressure levels as the observations and averaged vertically in the same manner. As a rough quality control check, superobservations with increments that are more than seven times the observation error are rejected. This condition can be triggered by either large observation errors or large model errors, and prevents unrealistically large updates to the assimilation system.

#### 2.3.3 Inflation and ensemble design

Background error variances are calculated from the ensemble; ideally, the ensemble spread should accurately represent uncertainty in the background field. Ensemble members should capture growing unstable modes of the atmosphere (Greybush, Kalnay, Hoffman, & Wilson, 2013); however, some parts of the Martian atmosphere are principally forced by aerosol heating, and the uncertain quantity is aerosol distribution. Therefore, the magnitude of the dust opacities and water ice cloud radiative properties is varied among the 16 ensemble members. Dust opacity increases uniformly from 0.7 to 1.3 times the amount specified by the tracers across the 16 members; water ice cloud radiative properties are multiplied by a scaling factor that alternates from 0.1 to 0.3 to 0.5. Finally, the background ensemble spread, which is typically underestimated because the data assimilation system does not account for model error, is increased. Spatially varying adaptive inflation (Miyoshi, 2011) is used to modify the ensemble spread to enforce the spread/skill relationship outlined in Desroziers, Berre, Chapnik, and Poli (2005) that ensemble variance plus the observation error variance matches the variance of the observation increments. The tuning parameter for the background spread standard deviation, which controls how quickly the adaptive inflation values change in time, is set at 0.04.

#### 2.3.4 Localization

The impact pattern of an observation upon the analysis is shaped by the structure of the background error covariance. In an EnKF, this is sampled from ensemble perturbations. Due to the limited ensemble size, these patterns are subject to sampling error. Localization assumes that correlations between distant points are due to sampling error, and smoothly truncates the patterns as a function of distance. Here, R‐localization is employed (Greybush, Kalnay, Miyoshi, Ide, & Hunt, 2011), with a half‐length of 600 km in the horizontal and 0.4 log P in the vertical. In the LETKF, temperature observations update temperature, wind and surface pressure state variables.

#### 2.3.5 Dust

Horizontal dust distributions are derived from the Mars Climate Database version 5 gridded dust scenarios (Montabone et al., 2015), which are kriged composites of multiple spacecraft dust sources, mainly TES column opacities and MCS profiles that have been extrapolated to the surface to derive an estimated column opacity. As in Kahre, Wilson, Haberle, and Hollingsworth (2009), the model equations for the lowest model levels (the boundary layer) include a source/sink term for dust that relaxes the model column opacities towards the observed column opacities. Otherwise, the three dust tracers are advected by the model winds, and the vertical profile is driven by advection and sedimentation.

#### 2.3.6 Special considerations

Special consideration must be given to Mars atmospheric phenomena such as thermal tides and CO_2_ condensation during assimilation. Zhao et al. (2015) found that a 6‐hr assimilation window caused a spurious enhancement of the thermal tides; this was corrected by using a 1‐hr assimilation window instead. Analysis increments of surface pressure are scaled globally to conserve atmospheric mass (Greybush et al., 2012). As TES observations fall below the CO_2_ critical temperature by several degrees (Colaprete, Barnes, Haberle, & Montmessin, 2008) which would lead to excess CO_2_ deposition, observations below the critical temperature are modified to match the critical temperature (Greybush et al., 2019). Wave‐0 and wave‐1 bandpass filters are applied to the mass (poleward‐most latitude) and wind (2 poleward‐most latitudes) fields, respectively, for geographically consistent increments near the polar singularity. A low‐pass filter is applied to the wind fields near the poles (third and fourth latitude circle), and a Shapiro low‐pass filter is applied to the analysis increments throughout to remove spurious high‐frequency noise.

## 3 DATASET DESCRIPTION AND FORMAT

### 3.1 Timekeeping on Mars

With a Martian sol (day) approximately equal to 24 earth hours and 40 earth minutes, and a Martian year approximately equal to 668.6 sols, a different timekeeping system is required for Mars. For EMARS purposes, hours are Martian hours, which are 24 equal divisions of the Martian sol. The convention of Clancy et al. (2000) is to label Martian years (MY) consecutively since 1955. Solar longitude (Ls), or areocentric longitude, values of 0°, 90°, 180° and 270° mark the Northern Hemisphere vernal equinox, summer solstice, autumnal equinox and winter solstice, respectively, and provide a convenient seasonal index. The Martian perihelion occurs at Ls 250.66. The Mars24 tool (Allison, 1997; Allison & McEwen, 2000) is used to convert MY and Ls directly to earth calendar dates. As the reanalysis temporal resolution is hourly, a calendar of Martian sols and hours is a practical time labelling. For timekeeping, EMARS follows the conventions of Montabone et al. (2015), which proposes a system of leap sols with years of lengths 669, 668, 669, 668 and 669 repeating successively. To address the Martian analemma, observations are assimilated using the local time attribute provided by the instrument teams. EMARS times should be interpreted as hour 12 corresponding to solar noon at longitude 0. Finally, ‘MGCM sols’ are labelled continuously since the MY22 perihelion, which predates all TES and MCS observations. A table of Mars time conversions is provided along with EMARS.

### 3.2 File naming and formats

EMARS version 1.0 spans MY24 Ls 103 to MY27 Ls 102 for TES (the complete TES period of record) and MY28 Ls 112 (the start of the MCS period of record) to MY33 Ls 105 for MCS. This represents approximately 3 TES years and 5 MCS years. EMARS was produced in separate ‘streams’ of approximately 1 Mars year in length; this approach has also been used for some earth reanalyses (Poli et al., 2016). Changes in stream occur at Ls 105, with the switch occurring at the start of the sol that contains Ls 105. Users can expect slight discontinuities at this point, although this point was selected to be at a time in the Martian year with reduced variability.

The dataset is provided in NetCDF format. NetCDF is a self‐describing file format (metadata is stored along with actual data) common in the atmospheric science community for model output, and tools for reading and writing are readily available online.

Each reanalysis file type is divided into 12 segments per year, corresponding to 30 degrees of Ls. This corresponds to file sizes between 1 and 5 GB, which are a compromise between too many small files and too large of an individual file. The total size for the complete EMARS dataset is estimated to be on the order of 2 TB.

With EMARS, we provide three types of files (Table 1): ‘analysis’ files, ‘background’ files and ‘control’ files. Analysis files represent the model restart files that have been directly updated by the data assimilation system. They therefore should be used for any direct reanalysis‐observation comparison studies, as the model state variables will be closest to the observations. However, the format is not as convenient, with variables stored on a ‘D‐grid’ (Arakawa & Lamb, 1977) and only the ‘T’ (temperature), ‘U’ (zonal wind), ‘V’ (meridional wind), ‘ps’ (surface pressure) and ‘Surface_geopotential’ fields provided. Background files are created from 1‐hr MGCM forecasts, using the analysis files as initial conditions. These are more convenient, as there are many additional diagnosed variable types and all are provided on a common grid. Control files are from a freely running MGCM (without data assimilation) using the same horizontal dust distribution and model settings as EMARS; format is otherwise identical to background files. For analysis files, the ensemble mean and ensemble spread (standard deviation) are provided. For background files, the ensemble mean and a representative ensemble member are provided. For control files, a representative ensemble member is provided.

**Table 1:** EMARS file types

File type	Data provided	Description
Analysis	Ensemble Mean, Ensemble Spread	Direct output from data assimilation; contains only updated state variables
Background	Ensemble Mean, Representative Member	Short‐term (1 hr) forecast from analysis; includes other model fields
Control	Representative Member	Direct output from model, no data assimilation employed

File naming conventions follow: emars_v[version number]_[file type]_[member type]_MY[Mars year]_Ls[starting Ls value]‐[ending Ls value].nc. The version number here is 1.0. File type can be ‘anal’ (analysis), ‘back’ (background) or ‘cntl’ (control). Member type can be ‘mean’ (the ensemble mean), ‘sprd’ (the ensemble standard deviation) or ‘memb’ (a representative ensemble member; here, member 008 which has the median amounts of dust and water ice cloud forcing). Two sample filenames would be the following:

emars_v1.0_anal_mean_MY25_Ls060‐120.nc.

emars_v1.0_back_memb_MY26_Ls300‐360.nc.

### 3.3 Description of variable types

The dimensions of the dataset in *x*, *y*, *z* and time are described in Table 2. The corresponding static variables for spatial and temporal extent are described in Table 3. For the horizontal coordinate system, values for latitude and longitude for the standard ‘A’ grid in which all variable types are co‐located, along with the ‘D’ grid variables used only for winds in analysis files, are included. The hybrid vertical coordinate is uniquely defined by a surface pressure field, as well as *a_k_* and *b_k_* coefficients that describe the pressure and sigma (terrain‐following) portions, respectively. Pressure at each interface *p_i* between two vertical levels *k* is given as.

$$p\_i_{k}=p_{\text{sfc}}*b_{k}+a_{k}$$

The pressure at the centre of the corresponding layer *p_k_* is given as.

$$p_{k}=\frac{p\_i_{k+1}-p\_i_{k}}{\ln(p\_i_{k+1}/p\_i_{k})}$$

**Table 2:** Dataset dimensions

Dimension name	Number of values	Description
lon	60	Longitude
lat	36	Latitude
pfull	28	Vertical level
phalf	29	Vertical level interface
time	~1,000	Time
lonv	60	Longitudes used for v‐wind in ‘analysis’ files, which are in staggered D‐grid
latu	35	Latitudes used for u‐wind in ‘analysis’ files, which are in staggered D‐grid

**Table 3:** Static variables used to define spatial and temporal extent

Variable name	Dimension	Units	Description
lon (lonv)	lon	deg	Longitude in degrees E, 0‐360
lat (latu)	lat	deg	Latitude in degrees N, −90‐90
ak	lev	Pa	Pressure coefficient for hybrid vertical coordinate
bk	lev		Sigma coefficient for hybrid vertical coordinate
pfull	pfull	hPa	Sample pressure levels, given a reference surface pressure
phalf	phalf	hPa	Sample level interface pressures, given a reference surface pressure
Surface_geopotential	lat, lon	m^2^/s^2^	Surface geopotential height
time	Time	Hours	Number of Martian hours since start of file
MY	Time	Years	Mars year, following Clancy et al. (2000)
Ls	Time	Deg	Areocentric longitude
mars_hour	Time	Hours	Hour of the Martian day
mars_soy	Time	Days	Sols after the last Martian vernal equinox, using the Montabone et al. (2015) calendar
macda_sol	Time	Days	Sols after the start of MY 24; used for comparison with MACDA
emars_sol	Time	Days	Sols after MY 22 perihelion; used by MGCM
earth_year	Time	Years	Earth year
earth_month	Time	Months	Earth month
earth_day	Time	Days	Earth day
earth_hour	Time	Hours	Earth hour
earth_minute	Time	Minutes	Earth minutes
earth_second	Time	Seconds	Earth seconds

Sample pressures at levels and interfaces are also provided, given a reference surface pressure. Note that in EMARS, levels are numbered from 1 to 28 going from the top of the atmosphere to near the surface. Height (above MOLA zero elevation datum) is provided at level interfaces; it can be calculated at level centres using the hydrostatic relationship. A variety of time variables are included to facilitate conversion between earth times, solar longitude and Mars calendars employed for EMARS and MACDA.

Table 4 describes the reanalysis variables found in ‘analysis’ files, whereas Table 5 describes the variables found in ‘background’ and ‘control’ files. Variables describe the thermal field, wind field, aerosol fields (both column and profile information) and surface fields.

**Table 4:** Variables in ‘analysis’ files

Variable name	Dimension	Units	Description
T	time, pfull, lat, lon	K	Atmospheric temperature
U	time, pfull, latu, lon	m/s	Zonal wind component
V	time, pfull, lat, lonv	m/s	Meridional wind component
ps	time, lat, lon	Pa	Surface pressure

**Table 5:** Variables in ‘background’ and ‘control’ files

Variable name	Dimension	Units	Description
t	time, pfull, lat, lon	K	Atmospheric temperature
u	time, pfull, lat, lon	m/s	Zonal wind component
v	time, pfull, lat, lon	m/s	Meridional wind component
ps	time, lat, lon	Pa	Surface Pressure
h	time, phalf, lat, lon	M	Height above MOLA zero elevation datum (not surface)
vap	time, pfull, lat, lon	kg/kg	Water vapour mass mixing ratio
cld	time, pfull, lat, lon	kg/kg	Water ice mass mixing ratio
o1	time, pfull, lat, lon	kg/kg	0.3 micron dust tracer mass mixing ratio
o2	time, pfull, lat, lon	kg/kg	1.2 micron dust tracer mass mixing ratio
o3	time, pfull, lat, lon	kg/kg	2.5 micron dust tracer mass mixing ratio
opac	time, pfull, lat, lon	Pa^−1^	Aerosol opacity, normalized over pressure level
omega	time, pfull, lat, lon	Pa/s	Vertical velocity in pressure coordinates
lheat	time, pfull, lat, lon	K/s	CO_2_ latent heating rate
hrad	time, pfull, lat, lon	K/s	Radiative heating rate
ts	time, lat, lon	K	Surface temperature
dod	time, lat, lon	Unitless	Column dust visible opacity (not normalized to a reference surface pressure)
tod	time, lat, lon	Unitless	Target dust visible opacity, from Montabone et al. (2015) (not normalized to a reference surface pressure)
vod	time, lat, lon	Unitless	Total visible opacity from aerosols (not normalized to a reference surface pressure)
frost	time, lat, lon	kg/m^2^	Surface water ice
snow	time, lat, lon	kg/m^2^	Surface CO_2_ ice
wcol	time, lat, lon	kg/m^2^	Column water vapour
cldcol	time, lat, lon	kg/m^2^	Column water ice
od1	time, lat, lon	unitless	0.3 micron dust column visible opacity
od2	time, lat, lon	unitless	1.2 micron dust column visible opacity
od3	time, lat, lon	unitless	2.5 micron dust column visible opacity
stress	time, lat, lon	N/m^2^	Surface wind stress

**Figure 1:**
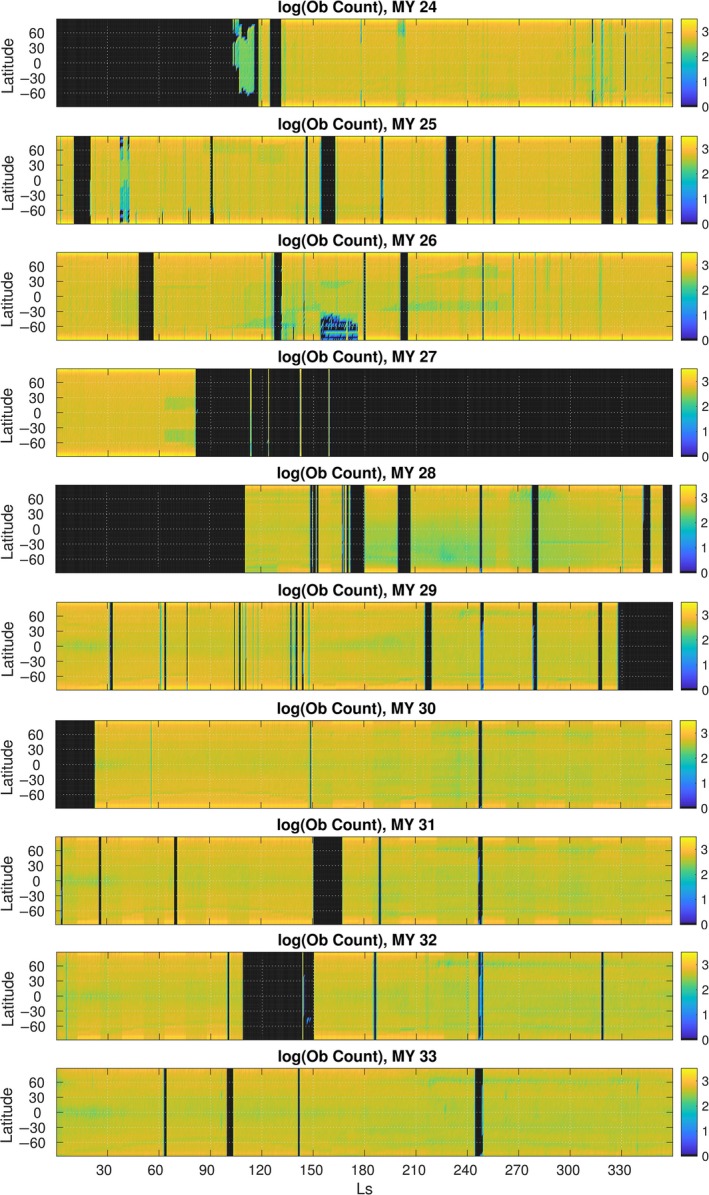
Plot of observation availability for TES and MCS by Mars Year and Ls. Counts are number of temperature superobservations, given on a logarithmic (base 10) scale

Finally, we have added a file ‘emars_v1.0_obscount’, which shows the number of daytime and night‐time temperature superobservations available for assimilation at each hour, which (like Figure 1) is helpful for determining when the reanalysis is constrained by observations.

## 4 DATASET ACCESS AND VISUALIZATION

The EMARS dataset is archived and available for download via the Penn State Data Commons, which is a publicly accessible, centrally managed, long‐lived resource available to Penn State University investigators (http://www.datacommons.psu.edu/). The Data Commons has the capability to generate a Digital Object Identifier (DOI) for datasets, as well as create sufficient, searchable documentation (metadata) for all hosted data. A landing page for the data can be found at ftp://ftp.pasda.psu.edu/pub/commons/meteorology/greybush/emars-1p0/a_landing_page.html, and the data can be accessed via: ftp://ftp.pasda.psu.edu/pub/commons/meteorology/greybush/emars-1p0/data.

**Figure 2:**
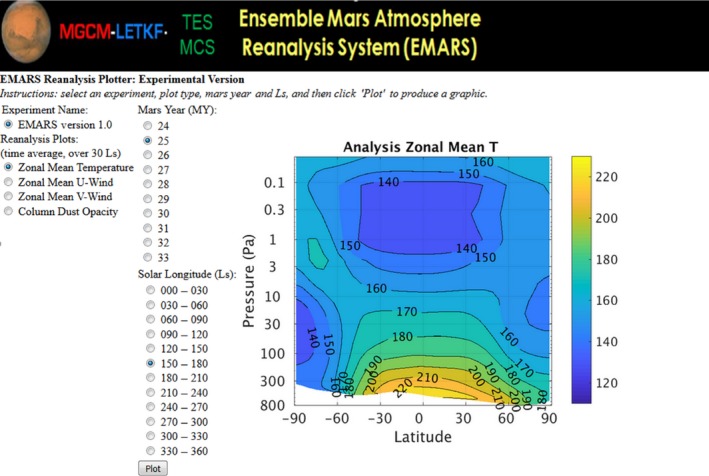
Sample screenshot from the EMARS plotter

**Figure 3:**
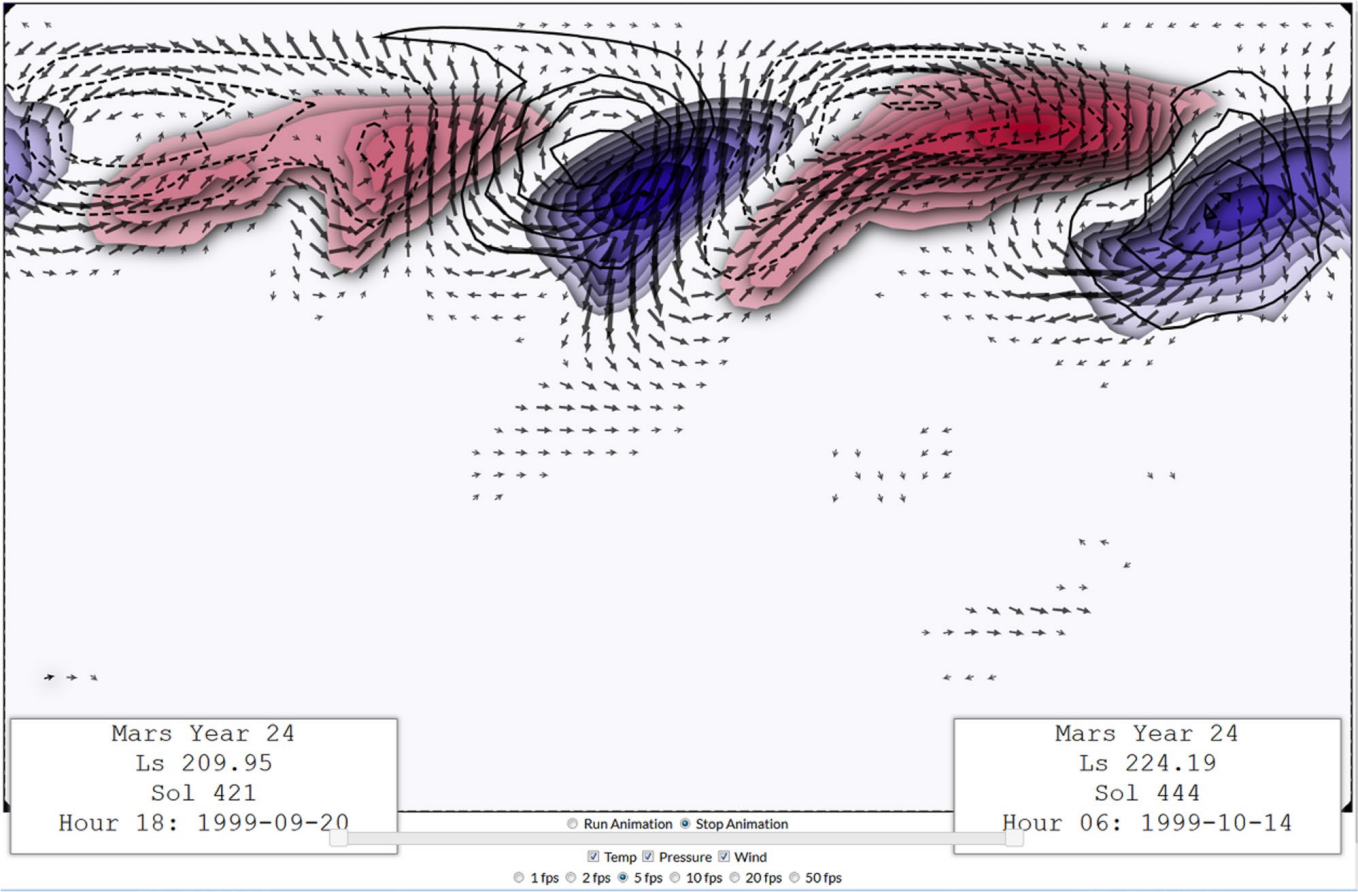
Sample screenshot from the EMARS animator depicting transient eddy temperature (colours), surface pressure (black contours; solid for positive, dashed for negative) and wind (vectors) anomalies

The Ensemble Mars Atmosphere Reanalysis System can be visualized in multiple ways. An EMARS plotter allows the visualization of seasonal average (over 30° Ls) statistics for key variables such as zonal mean temperature, winds and column dust opacity. Figure 2 shows a sample interface and image generated by the plotter. Synoptic states of EMARS, that is the state of the atmosphere at a specific instant in time, can be visualized as well. The EMARS plotter can be accessed at

http://www.meteo.psu.edu/~sjg213/emars_plotter/. Figure 3 shows a snapshot of an animation depicting transient eddies in EMARS; the video for the animation is available as supplemental material for the paper, and the methods for calculating the transient eddy fields are found in Greybush et al. (2019).

## 5 DATASET USE AND REUSE

Martian reanalyses, such as EMARS, have a variety of uses to the scientific community. A feature‐based evaluation of a reanalysis assesses the appropriateness of the dataset for the study of specific aspects of the Martian atmosphere. This complements a forecast‐based evaluation using tools favoured in the data assimilation community, such as examining short‐term forecasts minus observations and ensemble spread. Such feature‐based studies also provide the opportunity to advance our understanding of these phenomena, as well as encourage improvements to modelling, observations and data assimilation procedures to better represent them in reanalyses. The following paragraphs examine several such aspects, describe actual uses of the dataset in the study of that feature and provide recommendations for potential future use.

### 5.1 Zonal mean temperatures, circulation and polar vortex

Greybush et al. (2012) computed zonal mean temperature biases and root mean square errors between short‐term forecasts and (independent in time) TES observations. Biases were found to be small, and RMSEs were generally less than 5 K. The largest differences are in the vicinity of the sharp temperature gradients of the polar vortex. For MCS observations, the largest systematic temperature differences are above 40 km in altitude. In agreement with GCM simulations, in EMARS the global Hadley circulation has ascending air in equatorial regions (spring/fall) or the extratropics (summer hemisphere), and descending air aloft causing polar warming above the vortex; there are some differences in the exact orientation of this warming (McCleese et al., 2017). Waugh et al. (2016) examine polar vortices in both MACDA and EMARS, and reveal steep PV gradients near the poleward edge of westerly jets, and an annular structure in potential vorticity around the winter poles. Figure 2 and the EMARS plotter provide views of the zonal mean state of EMARS at various seasons and years.

### 5.2 Transient eddies

Transient eddies, or travelling waves, represent one of the largest sources of synoptic scale day‐to‐day variability in Martian weather. Greybush et al. (2019) review transient eddy seasonality, amplitudes and wavenumber regimes. Whereas the general features of synoptic states appear robust, details can be sensitive to modelling and data assimilation configurations. There are some systematic differences between seasonality and amplitudes of TES and MCS transient eddies, which may be due to instrument differences rather than interannual variability. TES eddies generally show a smaller ensemble spread around unique synoptic states than MCS eddies.

### 5.3 Thermal tides

Zhao et al. (2015) showed that data assimilation can potentially have a detrimental effect on the representation of thermal tides, with a six‐hour assimilation window causing a spurious amplification of the diurnal tide. The use of a 1‐hr assimilation window greatly improved tidal features in EMARS, with these features generally comparable to those of the control simulation. Navarro et al. (2017) pointed out that the global nature and forcing of the tides make them difficult for the assimilation to correct. The effective use of observations from multiple local times, as well as the vertical distribution of aerosol heating, to improve the representations of tides in reanalyses is still a work in progress.

### 5.4 Dust cycle

The horizontal dust distributions are constrained by the Montabone et al. (2015) gridded products, and a detailed evaluation of these distributions is contained within that work. Of note are systematic differences between TES and MCS opacities near the polar cap edges. While the vertical dust distributions in EMARS are subject to advection and sedimentation, lifting mechanisms for detached dust layers (Heavens et al., 2014) are not yet part of the MGCM, and this version of EMARS does not explicitly consider MCS vertical profiles of dust.

### 5.5 Water cycle

Zhao et al. (2015) demonstrated the improvement to reanalyses of the inclusion of radiatively active water ice clouds. This version of EMARS also includes an improved MGCM control simulation, with a water cycle spun up to more closely resemble the TES water vapour record (Smith, 2002). However, this version of EMARS does not explicitly assimilate water ice cloud opacities from TES and MCS. Therefore, the clouds in EMARS may be subject to GCM biases, such as thick cloud layers over the winter poles (McCleese et al., 2017). Assimilation of clouds may be challenging (Navarro et al., 2017) due to the role of tides in cloud formation (Benson et al., 2010; Lee et al., 2009) and errors in model representation of cloud physics.

### 5.6 CO_2_ cycle

While EMARS does not explicitly assimilate the locations of seasonal CO_2_ ice caps, informal comparisons with MOC observations and the Titus (2005) database show reasonable agreement. Precise tuning of a CO_2_ cycle to match lander surface pressure records, including local‐scale effects that are not well represented in a relatively coarse global model, can be challenging. While EMARS does not have explicit CO_2_ microphysics, CO_2_ latent heating reveals the locations of likely CO_2_ clouds over the winter poles.

### 5.7 Modelling studies and predictability

Mars reanalyses have potential uses for modelling studies, such as providing boundary conditions for high‐resolution regional simulations, trace gas estimation and transport, and facilitating the development of improved model physics to more closely match observations. The EMARS system, with additional development, could be used in a near‐real‐time setting for Mars numerical weather prediction. However, NWP for Mars is still in its early stages, and predictability is limited due to baroclinic/barotropic error growth (Greybush et al., 2013; Newman, Read, & Lewis, 2006) and forcing errors including suboptimal representation of aerosol heating (Zhao et al., 2015).

### 5.8 Engineering studies and spacecraft operations

Mars reanalyses are also useful for engineering studies for future Mars robotic and human exploration missions. An improved characterization of atmospheric conditions and their spatiotemporal variability that affect the orbital trajectories, aerobraking, aerocapture, descent and landing of spacecraft can allow for a smaller landing ellipse and open additional landing sites for consideration. Similarly improved characterization of dust opacity should benefit studies of solar power availability and surface operations aboard landers and rovers.

## 6 FUTURE VERSIONS

EMARS is a continually improving product, and innovations currently being developed by the EMARS group are expected to be included in future EMARS versions. These may include

- Improvements to the MGCM dynamics, including the switch to a cubed‐sphere geometry and increased horizontal and vertical resolution.
- Improvements to the MGCM physics, including CO_2_ ice cloud microphysics.
- Improvements to the LETKF data assimilation scheme, including the use of a hybrid variational–ensemble technique.
- Improvements to TES assimilation using interactive retrievals (Hoffman, 2010).
- Improvements to the vertical distribution of aerosols via assimilation of MCS dust and ice profiles (e.g. Navarro et al., 2017).

### OPEN PRACTICES

This article has earned an Open Data badge for making publicly available the digitally‐shareable data necessary to reproduce the reported results. The data is available at https://doi.org/10.18113/D3W375. Learn more about the Open Practices badges from the Center for Open Science: https://osf.io/tvyxz/wiki.
