# Supplementary material for: The Ensemble Mars Atmosphere Reanalysis System (EMARS) Version 1.0
Source: Geosci Data J. 2019 Aug 23;6(2):137–50. doi: 10.1002/gdj3.77 (PMC6919928; doi:10.1002/gdj3.77)
Supplement: Supplementary file 1 [file GDJ3-6-137-s001.docx]

Supporting information

The following supporting information is available as part of the online article:

Video S1.  Animation of transient eddies in EMARS.

Can be accessed at

ftp://ftp.pasda.psu.edu/pub/commons/meteorology/greybush/emars-1p0/videos/
